# Timing Determines Tuning: A Rapid Spatial Transformation in Superior Colliculus Neurons during Reactive Gaze Shifts

**DOI:** 10.1523/ENEURO.0359-18.2019

**Published:** 2020-01-02

**Authors:** Morteza Sadeh, Amirsaman Sajad, Hongying Wang, Xiaogang Yan, John Douglas Crawford

**Affiliations:** 1 York Centre for Vision Research and Vision: Science to Applications (VISTA) Program; 2 York Neuroscience Graduate Diploma Program; 3 Canadian Action and Perception Network (CAPnet); 4 Departments of Psychology, Biology, and Kinesiology and Health Science, York University, Toronto, Ontario M3j 1P3, Canada

**Keywords:** saccades, spatial transformation, superior colliculus, transformation, visuomotor

## Abstract

Gaze saccades, rapid shifts of the eyes and head toward a goal, have provided fundamental insights into the neural control of movement. For example, it has been shown that the superior colliculus (SC) transforms a visual target (T) code to future gaze (G) location commands after a memory delay. However, this transformation has not been observed in “reactive” saccades made directly to a stimulus, so its contribution to normal gaze behavior is unclear. Here, we tested this using a quantitative measure of the intermediate codes between T and G, based on variable errors in gaze endpoints. We demonstrate that a rapid spatial transformation occurs within the primate’s SC (*Macaca mulatta*) during reactive saccades, involving a shift in coding from T, through intermediate codes, to G. This spatial shift progressed continuously both across and within cell populations [visual, visuomotor (VM), motor], rather than relaying discretely between populations with fixed spatial codes. These results suggest that the SC produces a rapid, noisy, and distributed transformation that contributes to variable errors in reactive gaze shifts.

## Significance Statement

Oculomotor studies have demonstrated visuomotor (VM) transformations in structures like the superior colliculus (SC) with the use of trained behavioral manipulations, like the memory delay and anti-saccades tasks, but it is not known how this happens during normal saccades. Here, using a spatial model fitting method based on endogenous gaze errors in “reactive” gaze saccades, we show that the SC provides a rapid spatial transformation from target to gaze coding that involves visual, VM, and motor neurons. This technique demonstrates that SC spatial codes are not stable, and may provide a quantitative diagnostic marker for assessing the health of sensorimotor transformations.

## Introduction

Saccades and coordinated eye-head gaze shifts have been employed extensively to study the fundamental neural basis of sensorimotor transformations (Mays and Sparks, 1980; Wurtz and Albano, 1980; Gnadt et al., 1991; Freedman and Sparks, 1997a,b; Freedman, 2008; Sadeh et al., 2015; Sajad et al., 2015). As a result, the circuitry of the saccade system in humans is very well described (Fischer, 1986; Pierrot-Deseilligny et al., 1991a,b; Gaymard and Pierrot-Deseilligny, 1999; Munoz and Everling, 2004). Studies in non-human primates have revealed numerous additional details about the cellular and signal properties. For example, neurons that are activated during a gaze task in the superior colliculus (SC), frontal eye fields (FEFs), and lateral intraparietal cortex (LIP) can be categorized into populations of cells with “visual” responses (briefly delayed burst responses to a visual stimulus), “motor” responses (burst activity just before and after a saccade) or visuomotor (VM) responses, i.e., both visual and motor (Goldberg and Wurtz, 1972b; Goldberg and Bushnell, 1981; Bruce and Goldberg, 1985; Bruce et al., 1985; Munoz and Wurtz, 1995a,b; Freedman and Sparks, 1997a; Bisley and Goldberg, 2003; Gandhi and Katnani, 2011). The timing of these responses thus seems to imply a transformation between the visual and motor responses in the temporal domain, but demonstrating a transformation in the spatial domain has proven to be far more difficult and controversial.

Numerous studies have approached this question from the perspectives of (1) effector specificity (i.e., where and how is a general gaze command apportioned into separate commands for eye and head motion) and (2) what reference frames are used to code these variables (e.g., eye, head, or body centered). These questions remain controversial (Caruso et al., 2018), but the predominant consensus is that higher level structures like SC, LIP, and FEF primarily code target and/or gaze direction relative to initial eye orientation, with default effector-specific codes and reference frames developed downstream (Sparks, 1989, 2002b; Sparks and Hartwich-Young, 1989; Klier et al., 2001; Crawford et al., 2011; Steenrod et al., 2013). Consistent with this view, we recently quantified two-dimensional SC and FEF response fields (RFs) during naturally variable eye-head gaze shifts, and found that eye-centered coordinates provided better fits to the data than effector-specific codes and frames of reference (DeSouza et al., 2011; Sadeh et al., 2015; Sajad et al., 2015).

The more basic question of whether these structures employ a sensory code (specifying target location) or a motor code (specifying future gaze location) has also proven difficult to resolve, particularly in simple “reactive” saccades made directly to a target without delay. Various studies have suggested that neurons in SC, FEF, and/or LIP encode target location (Tian et al., 2000; DeSouza et al., 2011; Steenrod et al., 2013), the saccade command (Mays and Sparks, 1980; Freedman and Sparks, 1997a; Horwitz and Newsome, 1999; Knight and Fuchs, 2007; Marino et al., 2008), or some time-dependent combination of both (Sadeh et al., 2015; Sajad et al., 2015). This is especially difficult to test in reactive saccades because they typically are accompanied by little temporal separation between visual and motor responses, and little spatial separation between the direction of a visual stimulus and saccade direction (Mays and Sparks, 1980; Waitzman et al., 1988; Stanford and Sparks, 1994; Munoz and Everling, 2004).

Various other behavioral paradigms have been exploited to dissociate target versus gaze coding, but each poses its own interpretive challenges, when one attempts to relate their results to reactive saccades. A memory delay can be used to produce saccade errors that dissociate the sensory and motor vectors (Stanford and Sparks, 1994). In a recent variation of this approach, we tested SC and FEF responses before and after a memory-guided gaze shifts, and then determined if the resulting RFs produced best fits against target coordinates versus motor coordinates that accounted for variable gaze errors (Sadeh et al., 2015; Sajad et al., 2015). The best fits suggested that the visual response encodes target direction relative to initial eye orientation (Te), whereas the motor response encodes the final gaze direction relative to initial eye orientation (Ge). This transformation did not occur in a single discrete step, but rather proceeded through intermediate codes along a “T-G continuum,” finally shifting to G in pure motor cells active just before a saccade (Sajad et al., 2016a). However, such memory-delay paradigms introduce suppression signals, memory signals, and a memory-motor transformation. These signals might introduce the accumulation of noise, creating an apparent “transformation” that may not be present in simple reactive saccades (Stanford and Sparks, 1994; White et al., 1994; Ohbayashi et al., 2003; Barber et al., 2013; Hollingworth, 2015; Sadeh et al., 2015, 2018; Sajad et al., 2015, 2016a).

The anti-saccade paradigm produces an extreme visual-motor dissociation by training animals to saccade opposite to the target. Anti-saccade studies have shown that many cells in the SC, FEF, and LIP initially encode visual target direction, but then switch to coding saccade direction (Gnadt and Andersen, 1988; Gnadt et al., 1991; Groh and Sparks, 1992; Everling and Munoz, 2000; Russo and Bruce, 2000; Paré and Hanes, 2003; Zhang and Barash, 2004; Marino et al., 2008; Watanabe et al., 2009). However, anti-saccades require suppression signals and spatial transformations that are not present in reactive saccades (Bell et al., 2000; Everling and Munoz, 2000; Munoz and Everling, 2004; Coe and Munoz, 2017), and thus introduce “non-standard” transformations (Hawkins et al., 2013; Sayegh et al., 2014). Second, anti-tasks can also cause directional tuning to reverse in occipital cortex, suggesting a remapped image rather than a sensorimotor transformation (Cappadocia et al., 2017). Studies that have attempted to disentangle these alternatives suggest that both may occur, even across different neurons in the same cortical area (Gail and Andersen, 2006).

Finally, double-step tasks combine both a memory delay and dissociation of the visual and saccade vectors, by interposing another eye movement within the memory delay. In such tasks, populations of cells in the SC, FEF, and LIP are known to update their coding direction from the original visual stimulus to the final saccade motor output (Mays and Sparks, 1980; Walker et al., 1995; Tian et al., 2000; Zhang and Barash, 2004; Dash et al., 2015). In terms of understanding “standard” VM transformations, this technique combines both the strengths and above weaknesses of the previous two techniques. In some cases spatial updating involves a transformation from visual to motor cells (Zhang and Barash, 2004; Dash et al., 2015) but visual neuroscientists often describe remapping phenomena as a process of updating target direction (Duhamel et al., 1992; Tian et al., 2000; Nakamura and Colby, 2002; Joiner et al., 2017). And again, spatial updating and/or remapping clear depend on very different neural mechanisms (such as saccade efference copies) than simple reactive saccades.

In summary, each of the above approaches have their own purpose and merit, but also pose interpretive challenges if one wishes to transpose their results to reactive saccades. Further, they require extensive training in animals, which potentially might alter normal synaptic connectivity. Thus, it is important to establish if similar transformations occur in reactive saccades. Our previous study concluded that the SC primarily encodes target location during reactive saccades (DeSouza et al., 2011), but that study did not separate different cell types and was not temporally sensitive enough to track spatial coding changes within the burst. And as noted above, our studies that did show such a transformation used a delay paradigm (Sadeh et al., 2015; Sajad et al., 2015). Thus, to our knowledge, no previous study has established if a spatial transformation occurs during the SC burst that accompanies simple reactive saccades.

In the current study, we directly investigated whether the continuous neural activity present during reactive saccades shows the same spatial transformation that been shown in the memory-delay paradigm (Sadeh et al., 2015; Sajad et al., 2015). To do this, we used the memory paradigm to sort SC cells into visual, VM, and motor types, but then recorded from these same neurons during the reactive task, and analyzing their spatial content using a recently developed model fitting approach (Keith et al., 2009; DeSouza et al., 2011; Sadeh et al., 2015; Sajad et al., 2015). Further, we used a variant of our recent spatiotemporal analysis (Sajad et al., 2016) to test for a rapid transformation within the continuous burst present during reactive saccades. In brief, we used our “model-fitting” approach to test the 2D RFs of each neuron at various time intervals within the SC burst, and plotted each of these against various coordinate systems (shifted along the T-G continuum). We found that, in the absence of a memory delay, SC neurons produce a rapid spatiotemporal transformation from retinal to gaze coding, through a distributed transformation that appears to depend more on timing than cell type.

## Materials and Methods

### Animals and surgical procedures

The data were collected from two female monkeys (*Macaca mulatta*, M1 and M2; age, 10 years; weights, 6.5 and 7 kg) with a protocol approved by the institution’s Animal Care Committee in accordance with guidelines published by the Canadian Council for Animal Care. With similar surgical procedures as described previously (Crawford et al., 1999; Klier et al., 2001), the monkeys were prepared for long-term electrophysiology and 3D (horizontal, vertical, and torsional) gaze movement recordings. Each monkey was subjected to general anesthesia with 1–2% isoflurane after intramuscular injection of ketamine hydrochloride (10 mg/kg), atropine sulphate (0.05 mg/kg), and acepromazine (0.5 mg/kg). As previously described (Sadeh et al., 2015; Sajad et al., 2015), to minimize the collisions between experimental setup and microdrive/electrode, we implanted a vertically aligned unit recording chamber (i.e., with no tilt) placed 5 mm anterior and 0 mm lateral in stereotaxic coordinates, which allowed access to the left and right SC. This chamber angle and position were chosen to minimize collisions between the electrode/microdrive and the experimental setup during head movements, and to simplify the use of stereotaxic coordinates during recordings. The chamber was then surrounded by a dental acrylic cap, which was anchored to the skull with 13 stainless steel cortex screws. Two scleral search coils (diameter, 5 mm) were implanted in one eye of the monkeys to record 3D eye movements. Two orthogonal coils, which were secured with a screw on a plastic base on the cap, recorded the 3D head movements during the experiments. 3D recordings and analysis were performed as described previously (Crawford et al., 1999; DeSouza et al., 2011).

### Experimental equipment

We used a Pentium IV PC and custom-designed software to present stimuli, control behavior paradigms, send digital codes to a Plexon data acquisition system, and deliver juice rewards to the monkeys. Stimuli were presented on a screen 60 cm in front of the monkey, by use of a projector (WT600 DLP projector; NEC). Monkeys were seated on a custom-designed primate chair to have their heads move freely at the center of a 1-m^3^ magnetic field generator (Crawford et al., 1999), and a juice spout (Crist Instruments) was placed on the skull cap for computer-controlled delivery of the juice reward to the monkey’s mouth.

### Behavioral recordings and paradigms

All experiments were performed in head-unrestrained conditions. This was necessary for the preliminary general reference frame analysis that preceded this experiment (Sadeh et al., 2015). Here, target (T) and gaze (G) position in eye coordinates were the key parameters, but head-unrestrained recordings also had advantages here: comfort, minimum experimental manipulations (i.e., our task did not require heavy training or special measures to dissociate between parameters and mimics a natural reactive gaze shift more closely), normal system behavior (since the natural behavior indeed involves eye + head movements and gaze shifts do not normally occur with the head fixed), adequate range of gaze motion for testing large neural RFs (see below), and the tendency toward more prolonged neural activity, amenable to a more detailed spatiotemporal analysis (Keith et al., 2009; DeSouza et al., 2011). Conversely, 3D recordings and analysis were required for the proper transformation of T and G data to eye coordinates, to account for the significant torsional eye rotation and prominent non-linearities that occur in the head unrestrained gaze range (Tweed and Vilis, 1987; Crawford et al., 1999; Klier et al., 2003; DeSouza et al., 2011).

**Figure 1.**
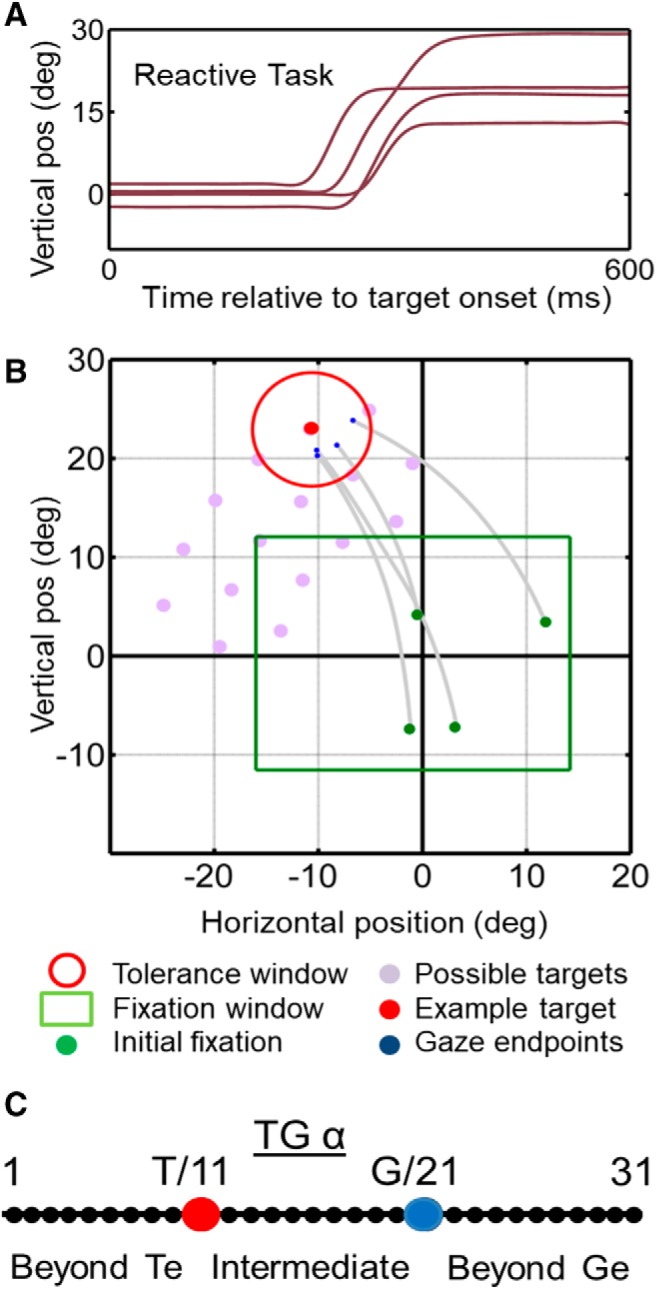
Reactive gaze task used for mapping neural receptive fields and fitting models. ***A***, Example traces of vertical eye position plotted as a function of time. ***B***, Two-dimensional gaze trajectories (gray lines) from the reactive task for an example target in monkey M2. Also shown are the range of initial fixation positions (green square), the tolerance window (red circle), and the other possible targets used in this experimental session (gray circles) to map a neuron’s receptive field. ***C***, The schematic illustrating the target gaze continuum concept, the distance between and beyond the target location and gaze are divided into 31 points, and the fit to neural activity is perform at each of the discrete locations to identify the best fit.

The primary behavioral condition used during our neural recordings was the reactive gaze shift task (Fig. 1), the fundamentals of the task are similar to those used in various studies previously (Deubel, 1995; Munoz and Wurtz, 1995a,b; Brown et al., 2004a; Alahyane et al., 2007; Cotti et al., 2009; DeSouza et al., 2011). The spatial aspects of this task were optimized for the model fitting analysis described below, including the separation of different reference frames and more importantly here, T from G coding. Animals were trained to begin each trial by fixating a central position (green circle with radius of 0.5°, tolerance window of 2–5° radius), with a location that randomly varied within a predetermined square range approximately equal to the cell’s RF size (this is not a tolerance window, merely a range of possible initial fixation positions), for 900–1000 ms (randomly varied interval). Simultaneous with initial fixation point disappearance, serving as the go signal, a target (red circle with a size of 0.5°) was presented in the periphery for 125 ms, brief enough to ensure no visual feedback after the completion of the gaze shift. The location was previously determined from preliminary RF mapping. Animals were then required to make a gaze shift toward the briefly flashing stimulus and fixate on it for 200 ms to receive juice reward. To spatially separate targets from gaze coding, we designated a tolerance window of 6–12° (diameter) for gaze endpoint inaccuracies (errors) around the locations of the targets, which resulted in a naturally-generated spread around the targets (Fig. 1*A–C*). This variable error is the basis of our analysis method Sajad et al. (2015).

A standard round reward window was used to reward monkeys for coming within a certain distance of the target, without biasing for direction. The shape of the initial range is somewhat arbitrary, so long as it provides enough variance in initial gaze position to separate space-centered versus eye-centered models. In the simulations used to develop this method, we found that a range approximately equal to the RF size is ideal (Keith et al., 2009) . Since we cannot a priori know the exact shape of the RF, and the exact shape is not important, we just started with a square and adjusted overall size for each neuron.

In addition, we recorded the same neurons in a memory-delay task. This was identical to the reactive task, except with a memory delay of 400–700 ms during which the animal had to maintain fixation before making a saccade. These results were analyzed previously (Sadeh et al., 2015) and are only used here to distinguish different neuron types. A more detailed description of eye-head kinematics in this task was described previously (Sadeh et al., 2015, 2018); here, we focused on gaze kinematics relative to target location.

### Trial definition and inclusion criteria

The beginning of a trial was marked by the appearance of the initial fixation point. The beginning of the gaze saccade was defined as the instant when its velocity exceeded 50°/s, and its end was at the time when its velocity decreased to 30°/s. We excluded trials on the basis of spatial and temporal criteria. First, trials in which the directions of the gaze shifts were completely unrelated to the direction of the target (e.g., opposite direction) were removed. In other words, saccades that were so inaccurate that monkeys might not have been attending to the task. For objective inclusion criteria of accurate and attentive gaze shifts we performed the following: we obtained the regression between errors in gaze versus retinal error (the retinal angle between the fovea and the target at the initial eye position before the gaze shift), and removed trials with gaze error exceeding two standard deviations from this regression line. Furthermore, every trial was visually inspected, and any trial in which the gaze shift consisted of multistep saccade was excluded. For each neuron, we required successful performance for at least 80% of total trials (mean ± SEM trials = 178 ± 16), and at least seven successful gaze shifts toward each target location (with a possible maximum of 15), after excluding erroneous trials. All included trials were considered for analysis irrespective of whether or not the monkey received a reward after the trial. Also, only neurons for which a sufficiently wide range of the RF was sampled were used for analysis. To enforce this criterion, during off-line analysis, and after applying exclusion criteria on trials and behavior, we checked to make sure that (in addition to having at least seven trials for each target) these targets covered a range that modulated the cells from minimal activity to peak activity, i.e., either surrounding the “hot spot” in closed RFs or including the largest testable rise in open RFs. Note that RF slope matters more than flat areas like plateaus or flat valleys, because these are the areas where our analytic method is most sensitive to (see below, Fitting spatial models against neuronal RFs). We used predetermined bin sizes of 16-ms width to generate spike density plots.

### Neural recordings

We recorded extracellular activity from the left and right SC with tungsten microelectrodes (FHC). The electrode was inserted through a guide tube, which was controlled by a hydraulic microdrive (MO- 90S; Narishige International). Isolated signals were amplified, filtered and stored for off-line sorting with the Plexon MAP system. The SC was identified according to criteria published previously (DeSouza et al., 2011; Sadeh et al., 2015). The steps of SC identification and confirmation are identical to those explained previously (Sadeh et al., 2015). The memory-delay saccade task was used to dissociate between visual and movement related activities and categorize cells into visual, VM, and motor neurons.

To categorize these different types, we used a burst frequency threshold relative to baseline. Specifically, visual neurons were defined categorically as cells that showed a robust burst of activity (firing rate cutoff of >50 spikes/s above the baseline at peak) after the stimulus presentation (Goldberg and Wurtz, 1972a,b; Mays and Sparks, 1980). We used a window of 60 to 160 ms relative to stimulus presentation for analysis of neural activity in visual cells (Freedman and Sparks, 1997a; DeSouza et al., 2011; Marino et al., 2012; Sadeh et al., 2015). Motor neurons were defined as those that had 50 spikes/s above baseline activity during the interval from –50 to +50 ms relative to saccade onset; with activity starting before the gaze onset, and that continued to fire ∼100 ms after gaze onset (Glimcher and Sparks, 1992; Munoz and Wurtz, 1995a,b; Gandhi and Katnani, 2011). Neurons that met both criteria were classified as VM. The timing of peak of the activity was determined by careful visual inspection using spike density plots.

We also used a VM index (VMI) metric to quantify the relative distributions of visual versus motor vigor in a continuous fashion. [VMI = (motor spike count – visual spike count)/(motor spike count + visual spike count), motor spike count was counted in ±50-ms window relative to gaze onset and visual spike count was counted in time window of 60–160 ms (relative to target or stimulus onset) to quantitatively separate these based on our previously published memory-delay task data (Sadeh et al., 2015). This gave a score, where –1 is a purely visual neuron and +1 a purely motor neuron.]

Compared to the categorical classification (based on 50 spikes/s > baseline), the VMI calculation resulted in ranges of –0.83 to 0.098 for visual neurons, VM neurons ranged from –0.84 to 0.71 and the pure motor neurons had VMI values from 0.2 to 0.74. Note that the VMI calculation was based on average firing rate within the window, whereas the firing rate cutoff was based on the first observed peak of activity within the defined range. When we refer to “number of spikes” below, this refers to number of action potentials in these defined temporal windows, also we use neural activity and burst interchangeably to refer to the same concept of high frequency of action potentials. The variability of VMI and differences in categorization of neurons resulted from the differences in vigor and characteristic of cell discharges depending on their class (for example buildup activity vs burst type), which we did not consider as a sub-population in our analysis. Also, the VMI is a measure of the relative strength of the visual and motor activity so it can result in some wide fluctuations in values, especially for lower firing rates. We therefore used the 50 spikes/s > baseline criteria for classification because it is simple, immune to relative influences, and ensured good signal-to-noise ratios for our analysis.

**Figure 2.**
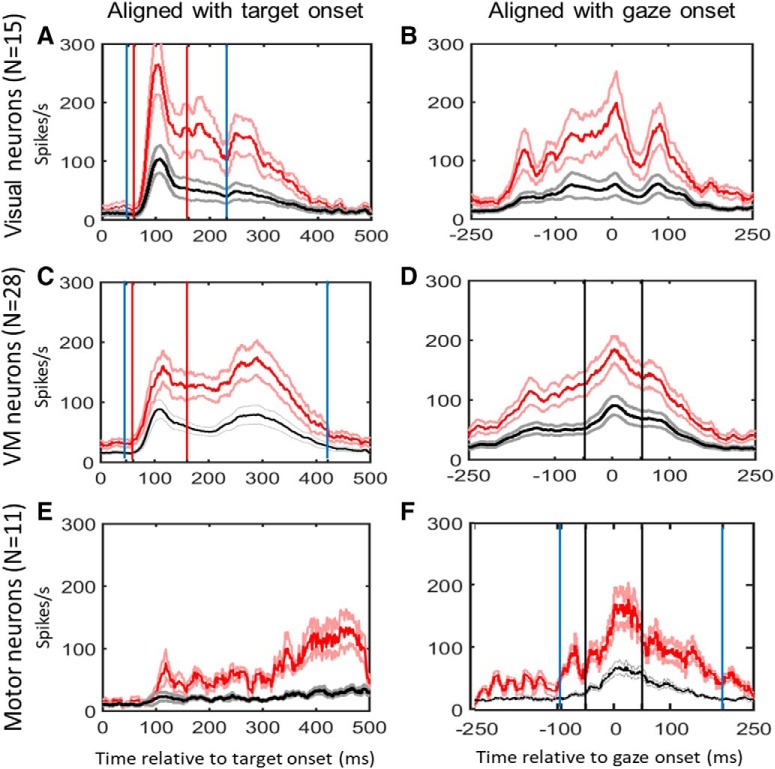
Mean spike density plots for our visual neurons (***A***, ***B***, *N* = 15), VM neurons (***C***, ***D***, *N* = 28), and motor neurons (***E***, ***F***, *N* = 11) during the reactive task. These sub-populations were identified using the memory delay task (Sadeh et al., 2015), not shown here. Data are aligned with stimulus onset (left column) and gaze movement (right column). These data were averaged across all data that passed our exclusion criteria. Red lines represent spike density plots derived from the “top 10%” trials in the reactive task (±SEM, light red lines), generally corresponding to the RF hot spot, and the black lines are derived from the average firing rate across all trials (±SEM, gray lines). Solid blue vertical lines indicate the average temporal analysis window for the full burst analysis, whereas red and black vertical lines indicate the time intervals sued for the fixed-window analysis in visual and motor activities, respectively.

**Figure 3.**
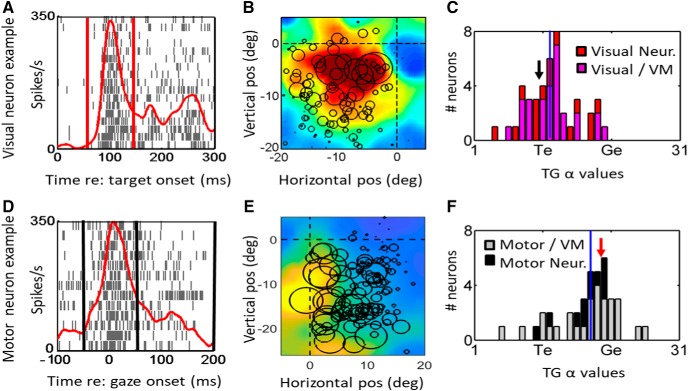
Shift of spatial representation from near Te in the target-aligned fixed window analysis (top row, ***A–C***) toward Ge in saccade-aligned fixed window analysis (bottom row, ***D***, ***E***) of reactive task data. Each row shows the raster/spike density plot (left column) and best fit RF (middle column) for an example neuron, followed the distribution of T-G α values of full population (right column). The rasters (***A***, ***D***) show spike trains and spike density plots for the top 10% trials toward the hot spot of the neuron. The center of circles in the RF plots (***B***, ***E***) represent the location best fit along the TG continuum in eye frame of reference and the diameter of circles are proportional to the firing rate of the neuron for that given trial. The heat map in the background indicates the RF fit to that data in this coordinate system. For the visual response population (***C***) both visual (red bars) and visual activity of VM neurons (pink bars) are included. For the motor response population (***F***), the motor activity of VM neurons (gray bars) and motor neurons (black bars) are shown. The red/black vertical lines in the raster plots (***A***, ***D***) represent the fixed visual/motor temporal windows, respectively. The black vertical lines in the histogram plots (***C***, ***F***) represent the median T-G α values and the location of T-G value for the representative example is indicated by the red arrow. The cluster of the distribution of visual fits (***C***) is closer to Te, whereas the cluster of motor fits (***F***) is closer to Ge. Note that the shift from the mean T-G values in the visual activity histogram (***C***; mean = 12.2) is significantly different (unpaired two-tailed *t* test, *p* = 0.0001) from the mean in the motor activity T-G histogram (***F***) mean (17.4).

**Figure 4.**
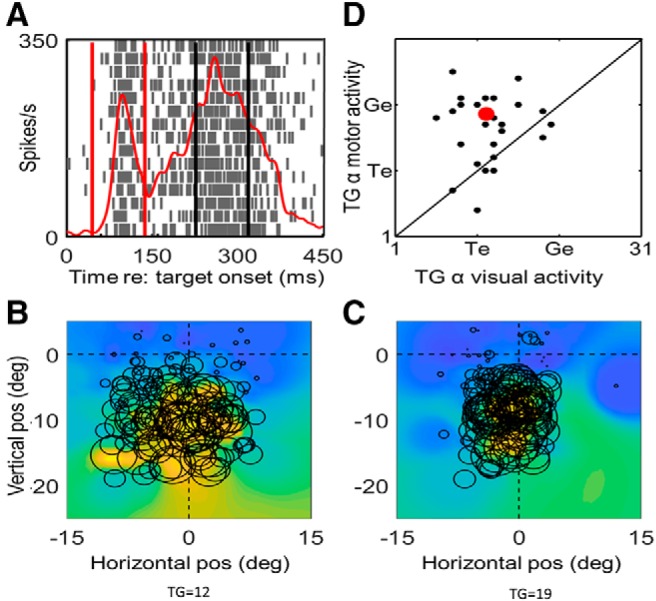
Shift from Te to Ge coding within VM Neurons. ***A***, Raster/spike density plot of a representative VM neuron aligned on target onset, showing fixed visual window (red lines) and average location of fixed motor window (black lines). This is followed by the best RF fit plots for the fixed (***B***) visual and (***C***) fixed motor activities. The circles on ***B***, ***C*** represent the location of target. ***D***, The scatter plot of differences in T-G α values of visual (*x*-axis) and motor (*y*-axis) of VM neurons (black circles) relative to the equality diagonal line. The average of the T-G α values in represented by the red circle and the representative example shown in ***A–C*** is indicated as the red circle. Most neurons lie above the line which indicates that there is a transition from coding for target location in the visual activity to gaze end location in the motor activity within the individual VM neurons. This shift was significant (paired two-tailed *t* test, *p* = 0.001).

**Figure 5.**
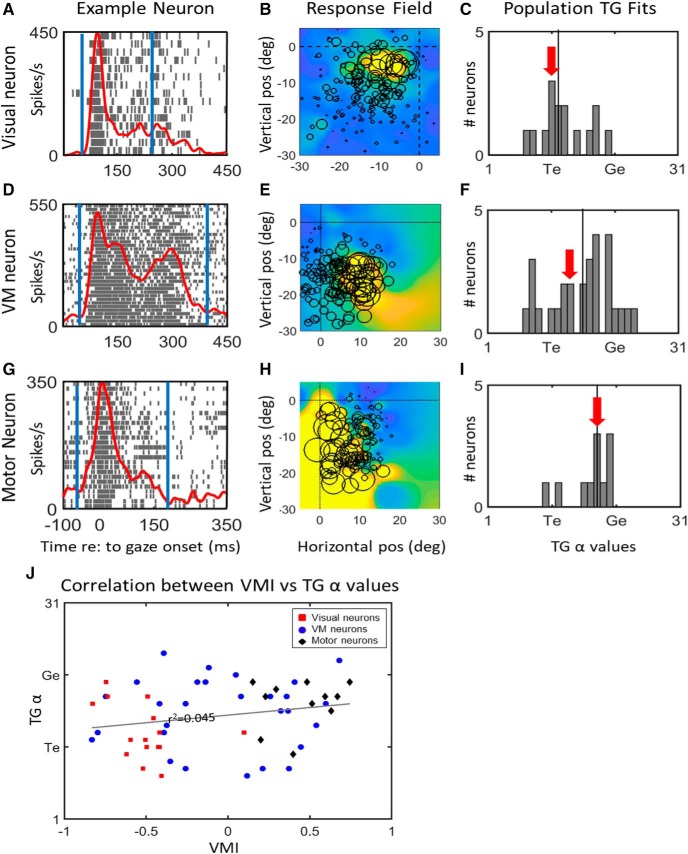
***A–I***, The T-G α value distribution for full burst analysis of visual (top row 1, ***A–C***), VM (row 2, ***D–F***), and motor (row 3, ***G–I***) neural populations in the reactive task. Each row shows an example neuron’s spike density plot/raster (column 1) and receptive field (column 2), and then a frequency histograph of best T-G fits for all neurons in that population. Spatial fits were made for each neuron using data derived the entire duration of task-related neural activity (between blue vertical lines in left column), aligned on stimulus onset. The vertical line in each panel of the right column indicates the median of the T-G α values and the red arrow indicate the T-G value of the representative example. ***J***, T-G α values plotted as a function of VMI for each neuron population. All neuron categories exhibit a weak, non-significant correlation: visual neurons are represented by red squares (*r* ^2^ = 0.1492, *p* = 0.155), VM neurons by blue circles (*r* ^2^ = 0.0012, *p* = 0.86) and motor neurons by black diamonds (*r* ^2^ = 0.041, *p* = 0.55). The overall correlation across all neurons (indicated by the gray correlation line) also leads a weak (*r* ^2^ = 0.045) non-significant (*p* = 0.123) correlation between the two variables.

**Figure 6.**
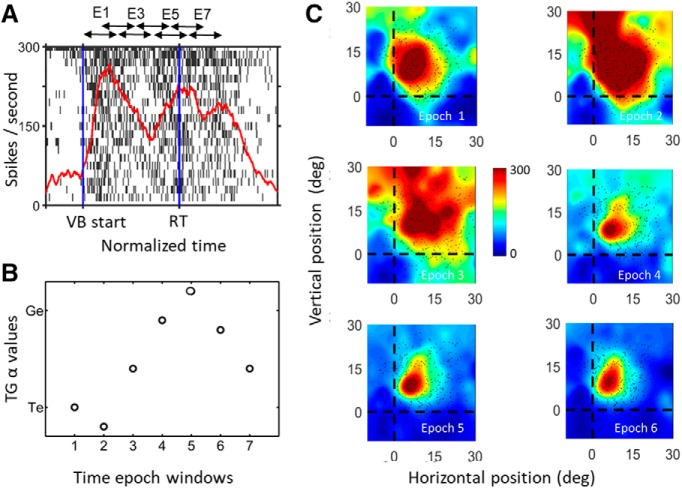
Spatiotemporal analysis in one example neuron. ***A***, Action potential raster plot and spike density plot of a representative VM neuron during the reactive task. The spike density plot (thick red line) was derived from the trials with the top 10% of activity (*N* = 19), i.e., when the target was presented at the hot spot of the RF. The dark blue vertical lines indicate the normalized sampling window of the VM burst, with first blue line indicating the start of the visual burst (VB) and the second blue line indicating the normalized reaction time (RT). The *x*-axis represents the normalized time relative to target onset, and the *y*-axis represents the firing rate. The double headed arrows on top of the raster plot indicate the semi-overlapping time windows which were used for the RF and T-G value analysis shown in ***B***, ***C***. These sampling windows were normalized according to the duration of the action potential (–370–200 ms from VB onset to movement onset) to yield seven semi-overlapping windows with equal time periods. ***B***, T-G continuum values plotted as a function of their sequence through time (1–7). In this case, there is a rise from T toward G over the first five steps followed by a slight reversal. The details of these patterns varied across neurons. ***C***, RF fits for the activity from time windows 1–6, plotted in the best fit reference frame along the target-gaze continuum (epoch 7 looked the same as 6). The dots indicate spatial positions of the targets in this frame for each trial and the color heat map (blue = low activity, red = high activity).

**Figure 7.**
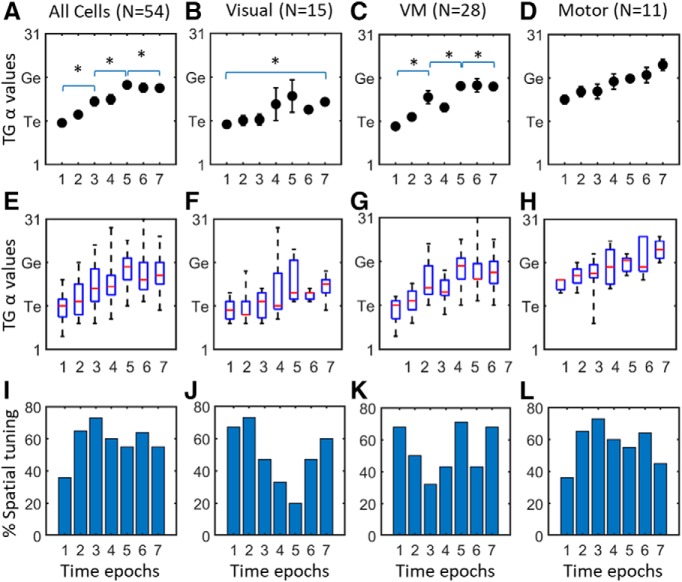
Spatiotemporal analysis in entire SC neuron population (column 1) and each subpopulation (columns 2–4). Top row (***A–D***) shows the mean T-G α values (*y*-axis) of each temporal window of analysis (*x*-axis) with SEM bars, the middle row (***E–H***) shows the median values (red bars) as well as first and third quartiles (blue bars) of T-G α values (y axes) for the same data, and the bottom row (***I–L***) shows the percentage of cells in each time epoch that showed significant spatial tuning. The entire neuron population (column 1, *N* = 56), showed a progressive shift in each step from more Te related coding in the earlier visual activity to more Ge related as the activity becomes closer to gaze onset. The visual neuron population (row 2, *N* = 15) which showed a predominantly preference in coding for target especially in earlier windows with a non-significant shift toward intermediate T-G α value later in its activity (one-way ANOVA *p* = 0.402). The VM population (row 3, *N* = 28) showed a significant shift in T-G α values (one-way ANOVA *p* = 0.0001). The motor population (row 4, *N* = 11) started at a more intermediate T-G value and showed a non-significant shift toward G (one-way ANOVA *p* = 0.48). The significant differences between time epochs -pointed at by the end tips of the brackets (*p* < 0.05) are indicated by asterisk (*). However, as described in the text, there was no significant difference between these three patterns. Note that for the results shown in Figure 5*A–H*, the T-G values were included in the analysis only if the neuronal activity showed spatial tuning for that given analysis window.

The temporal windows that we used for analysis of bursting activity in the reactive task are illustrated in the results section (Fig. 2). For some analyses (Figs. 3, 4), we used a fixed window of +60 to +160 ms relative to visual target presentation for visual activity (shown as red vertical lines) and –50 to +50 ms relative to saccade onset (shown as black vertical lines). For other analyses (Figs. 5, 6), we considered the entire burst duration of the neurons (windows shown as blue vertical lines). The selection of “full burst” time windows was based on visual inspection (DeSouza et al., 2011) but validates this by an objective approach (see below). The average range of the selected temporal analysis window (aligned on stimulus onset) was 342 ± 62 ms. Although the majority of the data were analyzed with objective measures, here, we aimed to use visual inspection of data to show that the current, more sophisticated analysis provides a more nuanced picture of the data than the “one best model” result we reported in our earlier findings (DeSouza et al., 2011). For visual neurons the full duration of burst was defined as the time which the activity increases above 50 spikes/s after the stimulus presentation to a point at which the activity considerably declines (as detected by visual inspection). This window was on average from +48 ms (start, ±14) to +231 ms (end, ±32) relative to visual stimulus onset. For VM neurons the average range of the entire burst was +47 to +421 ms relative to visual stimulus onset SD = (11 and 38 respectively). Note that these values are different from memory guided task. We also aligned VM neuron activity with the gaze onset and performed the similar full burst analysis, but found no significant change in the results. For motor neurons the average range was –94 to 194 ms relative to saccade onset (SD = 22 and 33, respectively). Finally, for Figures 6, 7, we performed a stepwise analysis of the entire duration of individual neuron activities broken down into smaller time windows to investigate how spatial coding changed through time (see Spatiotemporal analysis approach below).

To report burst onset and duration results, and validate the visual inspection-derived windows used for some analyses, we used an objective approach developed and reported by Legéndy and Salcman (1985) and applied to neural recording data (for identifying burst onset) by Hanes et al. (1995) as well as Thompson et al. (1996). In brief, this approach assumes that spike activity behaves in a Poisson manner, and for every trial the periods of spiking activity in which the spikes are too close in time are calculated, such that these periods of spiking are those surpassing that described by chance alone (referred to as “surprise”). Here, we used the same approach and calculated all the bursts in the entire spike train, and then applied this method to determine the onset of each burst. In addition, we also calculated the mean discharge rate -determined by number of spikes from the period which fixation on the initial position starts, to 20–150 ms, which is a period which the start of visual burst definitely falls within-. Once we determined the burst onset time for every trial, we then obtained the average and standard deviation of the burst onset time estimate. We used the visual inspection method to define the outer bounds for our spatiotemporal analysis (see below) because it led to better signal-to-noise ratios, but both methods yielded similar results.

### Fitting spatial models against neuronal RFs

Our general model-fitting method was fully described in Keith et al. (2009) and has subsequently been employed in several previous neurophysiological studies (Keith et al., 2009; DeSouza et al., 2011; Sadeh et al., 2015, 2018; Sajad et al., 2016a). The basic principle is to perform non-parametric fits against RF data plotted in various 2D coordinate systems (corresponding to various spatial models), and then determine which one yields the best fits (i.e., lowest residuals) compared to the actual data (see below for details). The method is conceptually similar to that employed by Platt and Glimcher (1998), except that we do not assume that the RFs are restricted to any particular shape. The coordinate systems used for these fits are based on physical geometric and behavioral measures taken from the laboratory. What separates (or “deconvolves”) these different spatial models is the natural variability of gaze behavior. The current gaze task (Fig. 1*B*) produced variability in initial gaze, eye and head position, variable contributions of eye and head displacement to the gaze shift, and variable gaze errors relative to target position. This in turn produced variability between 11 different coordinate systems derived from our 3D eye and head recordings: target direction in eye, head, and space coordinates, final gaze direction in eye, head, and space coordinates, gaze displacement in space coordinates, final eye position and eye displacement in head coordinates, and final head position and head displacement in space coordinates. The specifics of these models and methods are described in detail elsewhere (Sajad et al., 2015; Sadeh et al., 2015).

To determine which of these coordinate system models best described our neural data, we followed a statistical procedure that has also been described in detail elsewhere (Keith et al., 2009; Sajad et al., 2015; Sadeh et al., 2015). In brief conceptual terms, our analysis software did the following: first, for each neuron, the neural activity from each trial was plotted as a function of 2D position in each of the coordinate systems described above (e.g., target in space, gaze in space, etc.) Graphic examples of such plots are provided in each sub-section of the results below (where the circles indicate the number of spikes for a given temporal window, plotted in specific coordinate systems). Within each of these coordinate systems, we then constructed non-parametric RF fits to the data, using Gaussian kernels with bandwidths ranging from 2° to 15° (in the results figures, these fits are represented as color heat maps). The quality of the model fits to the data were then quantified by calculating the predicted sum of squares (PRESS) residuals for all trials (Keith et al., 2009). Briefly, the PRESS residual for each trial was obtained by: (1) eliminating that trial from the data, (2) performing a fit to the remaining data points as described above, and (3) obtaining the residual between the fit and the missing data point. This is done for every trial for each neuron. The overall predictability power of the model for the recorded data set was then quantified as the average of PRESS residuals across all trials for that neuron. The best spatial code was then defined as the model and kernel bandwidth that yielded the overall best fit (i.e., smallest residual) to the data for each neuron. It is noteworthy that this analysis is not influenced by systematic errors in behavior, but instead relies entirely on variability in the spatial relationship between positions in different models.

In our previous study, we performed these tests on an overlapping but larger population of SC neurons recorded during the memory-delay paradigm (Sadeh et al., 2015). This analysis showed a preference for coding target relative to initial eye orientation (Te) in most visual responses, and this preference was significant compared to all other models at the population level. In contrast, the best overall fit for the motor response was future gaze relative to initial eye orientation (Ge). This fit was significantly better than all head and space-centered models at the population level. Ge was not significantly better than fixed displacement models of the eyes and head, but these models were by previous head-unrestrained stimulation results (Klier et al., 2001). The dataset for the current study employed a majority sub-set of the neurons from the previous study, but tested instead in the reactive saccade task. In a preliminary analysis, we repeated the statistical tests from our previous study (Sadeh et al., 2015) using the same target and saccade-aligned temporal windows. Since the analysis, most neurons, and the results were the same we did not repeat this here, but overall, we found the following: a preference for Te for the visual response, and Ge for the motor response at the population level. Note that the difference between Te and Ge arises from variable gaze errors, which we enabled in the previous and current study by rewarding animals for placing gaze within a relatively large spatial window.

### T-G continuum analysis

Our previous analyses have suggested that a spatial transformation occurs between SC visual and motor responses, best represented at the population level as a shift from coding Te to Ge. to test this directly, we created a series of intermediate models along a T-G continuum (Sadeh et al., 2015, 2018; Sajad et al., 2015, 2016a). The physical basis of the T-G continuum is illustrated in Figure 1*C*, which shows the T-G continuum for an example trial. This continuum extends between, and beyond Te and Ge position for every such trial. The intermediate spatial models were constructed by dividing the distance between target position and final gaze position for each trial into 10 equal intervals and 10 additional intervals extended on either end. This results in a series of intermediate models (1–31), where Te falls at 11 and Ge falls at 21. We then performed non-parametric RF fits for each of these intermediate models, as described above. The intermediate value (here referred to as T-G α value) that provided the best fit was then used to describe the neuron’s spatial code along this continuum at a given point in time. For example, a T-G α value of 16 would indicate a code halfway between T and G. We could then perform population statistics on these fits, or use them to plot data (see results for examples). In our previous study there was a significant shift along the T-G continuum when the visual and motor response was separated by a memory delay. Our first goal here, was to test whether the same transformation occurs when there is no memory delay.

### Spatiotemporal analysis

We also aimed to test the time course of the T-G α shift, if any, to see whether it was discrete or continuous, and if this differed between different cell types. to do this, the T-G continuum analysis was applied to a series of time windows spanning the visual and motor neural activities (for details, see Sajad et al., 2016a). To account for the variable duration of neural activity across cells, we normalized the time between the onset of modulation and the time of gaze movement onset. Modulation onset was derived from spike density functions aligned on target onset (mean = 57 ms after target onset for V (Visual) and VM neurons, and 86 ms for motor neurons). Gaze movement onset was measured from behavior on a trial by trial basis. The duration between this these epochs was on average 231 ms (±74 ms, SD) across all trials. Each normalized raster was visually inspected to confirm the timing of the windows, relative to first modulation visual bursts, movement bursts and the peaks.

The interval widths for our fixed visual and motor windows are consistent with the literature (Wurtz and Goldberg, 1972; Sparks and Hartwich-Young, 1989; Marino et al., 2008, 2015; DeSouza et al., 2011; Bremmer et al., 2016; Sajad et al., 2016a; Sadeh et al., 2018) and were derived from separate visual and motor responses in the memory-delay paradigm, as shown previously (Sadeh et al., 2015). The intervals used for our new spatiotemporal method (Fig. 6) are based on those in our previous report (Sajad et al., 2016a), but the number of intervals was modified to fit the current dataset. The choice of number of windows was made to result in time windows closest to 100 ms. On average, seven time windows resulted in a separation of 92 ± 23 (SD) ms between the inner limits of our standard visual and motor temporal windows. Therefore, there was no overlap between these two windows.

These time-normalized epochs were then divided into seven half-overlapping windows, and firing rate was computed for each window (i.e., spikes/s; number of spikes divided by the sampling interval for each trial). The decision of number of windows to use, was based on the rough ratio of the duration of the visual response to memory-delay period to movement response, including a postsaccadic period starting from gaze onset. The majority of 6th and all of final (7th) time step corresponded to postsaccadic period starting from the onset of gaze shift. Because of the time-normalization process the sampling window width scaled with the duration between visual response onset and movement onset on a trial-by-trial basis. On the seven-step time-normalized scale, the visual burst on average lasted four steps (SD = ±0.63 steps), ending by the end of the fourth time step in 91.2% of trials. The sampling window width was on average 75 ± 8 (SD) ms and was no shorter than 47 ms for any trial, ensuring enough neuronal spikes for effective spatial analysis.

### Confirmation of significant spatial tuning (in neuron populations)

Since the results of our analysis approach are only considered valid if the sampled neural activity exhibits spatial tuning, we excluded data recorded for a given neuron and/or temporal window if it did not exhibit significant spatial tuning. To achieve this, we used an approach described in details before (Sajad et al., 2016a), in brief we randomly shuffled the firing rate data (number of spikes divided by duration of the sampling window) and plotted them over the position data corresponding to the best-fit model, and repeated this procedure 100 times to obtain 100 random RFs. The PRESS residuals of these random RFs (and their respective mean PRESS values) were then obtained after fitting the data (non-parametrically, using Gaussian kernels) with the same kernel bandwidth that was used to fit the best-fit model, resulting in a total of 100 mean PRESS residuals. If the mean PRESS residuals for the best-fit model (PRESS_best-fit_) was at least 2 SD smaller than the mean of the distribution of random mean PRESS residuals, then the sampled activity was categorized as spatially selective.

## Results

### General observations

We sampled 86 SC neurons during head unrestrained gaze shifts. Of these 86, we were able to record a complete data set from 74 neurons, spanning both sides of the SC in each animal. Of these 74 neurons, 54 met all of our inclusion criteria (31 from M1 and 23 from M2), including 15 visual, 28 VM, and 11 motor neurons (as Identified using the memory-delay task; Sadeh et al., 2015). We analyzed the latencies of the saccades to ensure that the gap between the stimulus and response was not in the range of the memory saccades that we studied previously (Sadeh et al., 2018). The mean latencies for saccades in the reactive task were 227.8 ± 2.7 (SEM) ms for animal one and 282.3 ± 5.3 (SEM) ms for animal two. Subtracting stimulus duration (125 ms), this leaves a gap of only 102–157 ms (mean, 129 ms) between the stimulus and the response. This clearly distinguishes our current results from the previous memory-delay paradigm, where the stimulus and response were separated by a 400- to 700-ms fixation delay, plus the additional reaction time required to produce a saccade in response to a go signal.

Figure 2 shows the activity profiles of each category of neurons (visual, VM, motor) during reactive gaze saccades to the top 10% RF hot spot (i.e., the region of the RF with the highest neural activity) data (red traces) and the full RF dataset (black traces). Each panel provides mean spike density plots (averaged across neurons ± SEM). Data are aligned both with target onset (Fig. 2*A*,*C*,*E*, left column) and when aligned with gaze onset (Fig. 2*B*,*D*,*F*, right column). Vertical red and black lines indicate the “fixed-window” visual and motor analysis windows, respectively, whereas blue vertical lines indicate the average duration of the full burst analysis. (Note that Fig. 2 shows average full burst durations for neuron populations; some neurons burst for shorter or longer durations but sum over the whole range, so the mean population spike density plots show a longer duration than the mean full burst windows.)

By definition (using categorical classification method and not the VMI here) visual neurons showed a much stronger target-aligned response than saccade-aligned response (Fig. 2*A* vs *B*), VM cells showed approximately equal responses (Fig. 2*C* vs *D*), and motor neurons showed much stronger saccade-aligned responses (Fig. 2*E* vs *F*).

The visual neuron population showed a strong initial peak of activity 88 ± 11 ms (mean ± SD) after the stimulus onset, followed by a smaller secondary peak of activity at 170 ± 15 (mean ± SD) ms (Fig. 2*A*). The large third peak ∼240 ms past stimulus onset (i.e., after the target onset) was likely residual motor activity (i.e., not excluded by our memory saccade-based population criteria) because it was absent in the memory-delay task visual response (Sadeh et al., 2015), and aligned closely with saccade onset (Fig. 2*C*). This was excluded from the visual full burst analysis, except in the stepwise temporal analysis shown below (Figs. 6, 7). The prolonged and delayed bursts were not seen in the memory-delay task which was recorded from the same neuron and reported previously (Sadeh et al., 2015), and may represent early motor preparation rather than a classical visual response, which become more prominent when stimulus and saccade onset are closer in time (Munoz and Wurtz, 1995a,b; Sparks, 1999; Dorris et al., 2007).

The VM population showed a first peak, when averaged across all VM neurons, 106 ± 9 ms after the visual stimulus onset (Fig. 2*B*) and a second peak (Fig. 2).

±3 ms after the visual stimulus onset (Fig. 2*B*) and a second peak (Fig. 2) 3 ms after saccade onset (Fig. 2*D*), separated by a short period (average 95 ± 12 SD ms) of sustained activity. Motor neurons showed a single peak of activity (22 ± 6 ms) after saccade onset (Fig. 2*F*). Henceforth, we will refer to the data from our fixed target and fixed saccade-related windows as “visual activity” and “motor activity,” based on their temporal profiles, but use our T-G continuum analysis method to quantify what spatial parameters these activities actually encode in different neurons and at different times.

From the Poisson burst analysis, we found that the average onset of the visual burst in visual neurons in the reactive task is 65.24 ms (SD ± 18.3 ms) relative to target onset with a range of 48.11–78.46 ms. For the VM neurons, the onset of visual burst was 68.18 ms (SD ±20.5) relative to target onset with a range of 54.71–84.79. The burst onsets of visual and VM neurons were not significantly different (*p* = 0.154, two-tailed unpaired *t* test).

### Spatial transformation between visual and motor responses

In our previous paper (Sadeh et al., 2015), we used fixed visual and motor window analysis in combination with a memory-delay paradigm to show that SC and FEF visual responses tend to code T, whereas the motor responses, following a brief memory period, tends to code G (in eye coordinates). As noted in Materials and Methods, our previous results (Sadeh et al., 2015) and preliminary analysis of the current dataset suggested that Te and Ge provided the best overall model fits for the visual and motor response populations, respectively. This suggests that a shift in coding occurs along the T-G spatial continuum during the SC burst associated with reactive saccades (see Materials and Methods; Fig. 2). To test this directly, we first performed a T-G continuum analysis on fixed visual/motor window analysis on the reactive task data. Note that these two temporal windows were each 100 ms in duration, and on average were shifted from each other (start-to-start) by 192 ± 23 ms, meaning that they were separated end-to-start by only 92 ± 23 ms. Thus, we were testing whether a significant spatial transformation from T toward G coding occurred over a very short period of time.

Figure 3 provides example rasters and fixed analysis windows (Fig. 3, left column) and RF fits (Fig. 3, middle column) for a typical visual cell (Fig. 3*A*,*B*, top row) and motor cell (Fig. 3*D*,*E*, bottom row). The visual neuron (Fig. 3*A*) showed a prominent peak, starting ∼80 ms after target onset and reaching maximum at 100 ms. Although it did not show motor activity in the memory-delay paradigm (Sadeh et al., 2015), here the prominent visual response was followed by smaller secondary and tertiary peaks often seen in the SC visual response (Hafed and Chen, 2016). The RF (Fig. 3*B*) is plotted at its best fit along the T-G continuum, and shows a “closed” response that peaks ∼5° down and left from the fovea/current gaze direction (as shown by the large circle data points and red hot spot on the fit. In contrast the motor response peaks around the time of the saccade (Fig. 3*D*), it shows a typical “open” RF with rising activity down and to the left (Fig. 3*E*), and gives the best fit at a point along the T-G continuum shifted more toward G.

The right column of Figure 3 provides frequency histograms that contrast the T-G α values for visual and motor window fits for our entire population of cells. The results of the visual window analysis are shown in Figure 3*C*. Overall, this yields a mean (12.2) and median (12) and distribution (SD ±4.2) that clearly clustered near T (11). There was no significant difference between the means of T-G α values for the visual population (red bars) and the visual response of the VM neurons within the same time window (pink bars; *p* = 0.8738, unpaired *t* test). In contrast, our analysis of motor activity (Fig. 3*F*) yielded an overall mean (17.3), median (18), and distribution (SD ±4.7) that was shifted toward the Ge model. Again, there was no significant difference between the distribution of the motor neuron responses (black bars) versus the motor response of VM neurons (gray) within the same time window (unpaired *t* test, *p* = 0.85). More importantly, there was a significant difference between the distributions of the visual (Fig. 3*C*) and motor (Fig. 3*F*) responses (*p* = 0.0001, unpaired *t* test). Note that in each RF the individual circle represents the firing rate of the neuron for that particular location of target or gaze end point and the diameter of the circles is proportional to firing rate.

Remarkably, this rapid shift in coding can be observed even within individual VM neurons, such as the example neuron with raster/spike density plot shown in Figure 4*A*, visual receptive field in Figure 4*B*, and motor RF in Figure 4*C*. To directly quantify if a T-G α shift occurs within VM neurons, we plotted the T-G α value from the motor window as a function of the value of the visual window for each neuron (Fig. 4*C*; TG α values of 12 and 19 for this particular example). Neurons with data points that lie above the diagonal line indicate a different preference of spatial coding in their visual versus movement related activities. The mean of T-G α values for VM neurons is also indicated by a red circle in Figure 4*D*, which shows that as a population there is a shift from target to gaze coding when going from visual to movement related activities in the VM neurons. Overall, the motor T-G α values for VM neurons were significantly different from their visual T-G α values (paired *t* test, *p* = 0.0001). Thus, a rapid transformation along the T-G continuum occurred between visual and motor responses, even within VM neurons.

This analysis suggests that the spatial code in SC neurons is not stable during a reactive task, particularly within VM neurons. However, it is not yet clear to what degree the overall visual- motor transformation is influenced by the spatial contributions of different neuron types at different times. This is not trivial to answer, given that visual cells by definition are active before motor cells, this classification scheme and timing will interact. Does this VM transformation occur because (1) neurons with early responses have a fixed T code whereas later motor neurons show a fixed G code, (2) because a distributed transformation causes a spatial shift in the code of late responses away from T, or (3) due to some combination of these factors? The first possibility (cell-fixed coding) does not seem compatible with our VM data (Fig. 4*D*), but we performed a more in-depth analysis to explore this in more detail.

### T-G continuum in the full burst of visual, VM, and motor cell

To investigate whether there is an overall difference in spatial coding between the three different neuron types (V, VM, M), that could be influenced by a fixed neural code in each cell type, we analyzed the full burst (Fig. 2) of each of the neuron types in our study. In a previous paper (DeSouza et al., 2011) a similar model-fitting approach was used on the full burst of SC neurons during the reactive task, but that study did not use a memory-delay task to classify different neuron types, and did not provide a T-G continuum analysis (only “cardinal” models such as Te: target relative to eye position, Ge: gaze relative to eye position, etc.). Based on that analysis DeSouza et al. (2011) concluded that the SC burst primarily encodes (Te), but the current analysis provides a more nuanced picture.

Figure 5 shows the full burst analysis for our visual neurons (Fig. 5,*A–C*), VM neurons (Fig. 5,*D–F*), and motor neurons (Fig. 5,*G–I*), respectively, showing an example neuron (Fig. 5, left column), its RF at the T-G α value of best fit (Fig. 5, middle column), and the frequency distribution of T-G α for each population (Fig. 5, right column). The entire combined population (data not shown) generated a T-G α value median of 16.5 (SD ±4.4), roughly in the middle of the T-G continuum (16). However, the distribution of individual neuron fits was quite broad and possibly clustered near T and G, perhaps suggesting the co-existence of different spatial codes. Visual neurons (Fig. 5*C*) were clustered toward Te (11), with a mean T-G α value of 13 (SD ±3.8), VM neurons (Fig. 5*F*) continued to show a broad distribution, with mean of 15.8 (SD ±4.9), and motor neurons (Fig. 5*I*) clustered toward G (21; mean: 17.9, SD ±3.3). However, these differences were not significant (Kruskal–Wallis ANOVA, *p* = 0.051). Overall, this full burst analysis shows that SC neurons show a broad continuum of spatial tuning between T and G during the reactive task, with non-significant trends of visual cells clustering toward Te, motor cells clustering toward G, and the distribution of VM cells spanning both.

Despite these overall tendencies, each sub-population showed a distribution of fits along the T-G continuum (Fig. 5*C*,*G*,*I*). To test whether this was due to variations in spatial tuning within cell types, we correlated (Pearson correlation) the T-G fit of these cells obtained from their full burst in the reactive task against their VMI obtained from the same cells in our memory-delay task (Sadeh et al., 2015). The overall relationship is shown in Figure 5*J*, with each sub-population coded for color. This yielded very weak correlations for visual (*r* ^2^ = 0.1492, *p* = 0.155), VM (*r* ^2^ = 0.0012, *p* = 0.86), and motor cells (*r* ^2^ = 0.041, *p* = 0.55). Even the entire cell population only showed little correlation between T-G α value and VMI (*r* ^2^ = 0.045, *p* = 0.123), suggesting that the relative size of the visual versus motor burst was not the main determining factor in the spatial codes in these cells.

### Spatiotemporal progression of VM signals in the SC

To test whether timing is the key factor in determining the spatial code in SC cells during our task, we examined the progression of spatial code through time for each neuron. Specifically, the entire activity of each of the individual neurons in each category was divided into seven time windows using a time normalization method to account for differences in duration of activity (see Materials and Methods), the resultant T-G α value was combined for each individual window in each of the neuron categories to investigate the temporal progression and transformation of spatial codes in each of the populations (Sajad et al., 2016b). In addition, this method allowed us to examine time windows around gaze shift in smaller time epochs and to investigate whether changes in analysis time window duration significantly impacts the results.

Figure 6 illustrates this analysis using an example VM neuron. Figure 6*A* illustrates that this neuron had multiple peaks of activity, including an initial visual peak, a strong secondary visual response, and a motor response. Figure 6*B* shows the corresponding RFs of the first six windows (each plotted using its optimal fit on the T-G continuum), showing how they progress through time. Figure 6*C* then shows these T-G fits as a function of time. Note that although these fits often “bounce around” for individual neurons like this example, especially near the start and end where spike rate is rising and dropping and confidence is thus lowest, they show a general trend to progress from near T to near G, as one can see in the next analysis. Note that the color background of the RF changes proportional to the firing rate for that given time epoch and thus results in change of appearance which should not be interpreted as changes in the RF size or shape.

To test the temporal shift in spatial coding at the population level, we first pooled all visual, VM, and Motor cells, and looked at their progression of T-G coding across the 388 ± 53 (SD) ms duration of their response, yielding six temporal shifts of ∼64 ms (Fig. 7, first column). Most neurons showed significant spatial tuning during most time steps (bottom row), and only these were used in the T-G α calculation. Figure 7*A*,*E* demonstrates the mean and median values with SD and SEM bars, respectively, for each of our seven normalized time windows, and Figure 7*I* shows the percentage of data that was spatially tuned in each window (and thus included in the analysis). The trend of these results suggests a continuous progression of target related coding indicated by T-G α values closer to the T model (i.e., T-G = 11) in earlier more visually related activity to gaze coding (values closer to T-G of 21) in the after activities which are temporally correlated with gaze onset. We compared the T-G α values in time windows 1, 3, 5, and 7 to exclude comparison between the overlapping windows using Kruskal–Wallis non-parametric one-way ANOVA test and found an overall significant difference (*p* < 0.0001) between the windows. We also found significant differences in T-G α value of window 1 (mean: 11.1) compared to the values of windows 3, 5, and 7 (means: 14.7, 19.6, and 18, respectively, and *p* < 0.01, *p* < 0.001, and *p* < 0.001, respectively, corrected for multiple comparisons), and a significant difference between the T-G α values for intervals 3 and 5 (*p* < 0.001). In other words, there was already a significant T-G shift ∼129 ms into the VM burst of the whole neuron population (Fig. 7*A*), which continued to progress for another ∼129 ms. Further, the relationship between T-G code and timing of the response yielded a very strong correlation (*r* ^2^ = 0.94, *p* = 0.00001, Pearson correlation).

### Timing versus neuron type

As noted above, timing and a cell classification based on visual-motor balance could interact or mask each other’s effects. As a result, cell type differences could look like timing differences and vice versa. To disentangle these effects, we divided our time analysis data into separate visual (Fig. 7, second column), VM (Fig. 7, third column), and motor (Fig. 7, fourth column) populations, they each showed similar trends, except that the visual population code plateaued before reaching G. Note that over the course of our seven time steps, the percentage of spatially tuned visual cells (shown in the bottom row) peaks around the time of the late visual response and fades toward the saccade, whereas spatially tuned activity held steady in the VM population and ramped up in the motor population. Testing within the three populations, there was a significant difference between first and seventh time steps in the visual neuron population (*p* = 0.03), and there was also a significant difference between the first and third (*p* = 0.01), first and fifth (*p* = 0.0001) and first and seventh (*p* = 0.0001) time steps in the VM neuron population (corrected for multiple comparisons). No significant changes in the T-G α values were observed between the time windows in the motor neuron population, but each population showed a significant correlation as a function of timing: visual neurons: *r* ^2^ = 0.6, *p* = 0.0006; VM neurons: *r* ^2^ = 0.81, *p* < 0.00001; and motor neurons: *r* ^2^ = 0.96, *p* < 0.00001 (Pearson correlation).

Based on visual inspection, there appears to be a slight upward shift (from T toward G) in these time-normalized plots from visual (Fig. 7*B*), to VM (Fig. 7*C*), to motor (Fig. 7*D*) populations. However, there was no significant difference between these plots (*p* = 0.53, non-parametric one-way ANOVA test). These results suggest that a similar spatiotemporal progression occurs across different cell types in the SC during reactive saccades, and that the difference in spatial coding across different cell types (Fig. 3) are primarily due to the relative timing of their responses, rather than fundamental differences in neuron properties.

## Discussion

The process of transforming the visual information into movement commands must occur for a successful and timely gaze shift (Mays and Sparks, 1980; Gnadt et al., 1991; Crawford and Guitton, 1997; Pouget and Snyder, 2000; Snyder, 2000; Crawford et al., 2011; Sajad et al., 2015, 2016a). Here, we found that the SC participates in a rapid transformation from target to gaze coding, even in the absence of a memory delay or other experimental manipulations. Further, we have shown that this does not primarily arise because of some fixed intrinsic code within different cell types (at least along the visual-VM-motor continuum) but rather because of a continuous temporal progression through all cell types. It remains possible that cell type also made a contribution, although this did not reach significance in the current dataset. To our knowledge, this is the first direct demonstration of an internal spatiotemporal transformation during simple reactive saccades.

### Evidence for a visual to motor transformation in the SC

One traditional view of spatial coding in the SC is it codes retinal error information received from retina and striate cortex, and simply relays this to the brainstem (Distel and Fries, 1982; Fries, 1984; Waitzman et al., 1988; Optican, 1995; Sparks, 2002a; DeSouza et al., 2011). Alternatively, it has been demonstrated that the SC (and other cortical gaze areas) can provide a visual-motor transformation for gaze shifts when the experimental task introduced a temporal or spatial separation between the visual stimuli and movement initiation (Gnadt and Andersen, 1988; Everling et al., 1999; Munoz and Everling, 2004; Sadeh et al., 2015; Sajad et al., 2015). Recently, the anatomic basis for this has been demonstrated at the level of SC microcircuitry, where the visual response is transmitted from dorsal to ventral layers, the delay response is similarly distributed, but motor response recruitment proceeds in the opposite direction (Massot et al., 2019).

However, it can be argued that the separation of visual and motor events required some experiments, influences the spatial code by means of changing the cognitive demands on the neural circuit. For example, in the case of anti-saccades, the encoding of target location by visual activity, and the gaze location by the motor activity is practically forced, and similarly when memory-related errors are introduced in the case of a the memory-delay task (Mays and Sparks, 1980; Stanford and Sparks, 1994; White et al., 1994; Goldman-Rakic, 1995; Miller et al., 1996; Brown et al., 2004b; Hollingworth, 2015; Sajad et al., 2016a). The behavioral variability that is accounted for in our G model fits to motor activity would reflect errors that have arisen within the SC motor neurons or its inputs (including feedback), whereas other unaccounted for noise in could be (1) noise that is present in individual cells but cancels at the population level, (2) signals that are not related to space, such as timing, attention, or motivation, or (3) noise and/or signals that only arise downstream from the SC motor output

The current study used a simple behavioral paradigm (reactive gaze saccade made directly to targets with no delay), combined with a sensitive model-fitting approach that can track spatial codes based only on endogenous error in the system. Based on the results of our previous study, which tested a wide array of spatial models in a memory-delay task (Sadeh et al., 2015) we focused on two models: Target in eye coordinates (Te) and future gaze position in eye coordinates (Ge), and used “T-G” continuum between these models to test the VM transformation. The results were clear, even in the short time span (192 ± 23 ms) between our visual and motor analysis windows there was a significant shift in coding across our entire population from T toward a G code. To our knowledge this is the first direct neurophysiological demonstration of a spatial transformation in the VM transformation for reactive saccades, in the absence of additional delays and trained transformations. Thus, this transformation cannot be attributed to exogenous suppression, memory, or top-down transformation signals, but must arise within the normal sensorimotor circuit.

### Spatial coding in different SC cell types: fixed or dynamic?

It has been a subject of debate whether the SC codes T, target location (Sparks and Porter, 1983; Waitzman et al., 1988; Sparks, 1989; Basso and Wurtz, 1998; McPeek and Keller, 2004) or G, future gaze location (Walker et al., 1995; Freedman and Sparks, 1997a; Everling et al., 1999; Horwitz and Newsome, 1999; Klier et al., 2001). In a previous study (DeSouza et al., 2011), we concluded that overall SC activity preferred a T code during reactive saccades. In light of the current study, this was likely due to a mixture of different signals and the use of cardinal Te and G models rather than the T-G continuum. The full burst analysis of our different cell populations (Fig. 5) revealed a continuum of T-G codes across all three cell populations, with a preference for Te in V cells, a distribution that equally spanned T and G in VM cells, and a preference for G in M (Motor) cells. To our knowledge, this is the first time that specialization of spatial coding across cell types has been demonstrated during reactive saccades.

This apparent specialization is generally consistent with findings from other paradigms, such as our analysis of SC activity in a memory-delay task (Sadeh et al., 2015). It also makes sense in terms visual cells presumably reflecting visual input most closely (Wurtz and Mohler, 1976; Wurtz and Albano, 1980; Moschovakis et al., 1988a), motor cells reflecting output (Sparks and Hartwich-Young, 1989; Miyashita and Hikosaka, 1996; Sparks, 2002a), and VM cells reflecting both as well as more complex influences. VM neurons are known to receive a more extensive range of inputs from other brain areas (Wurtz and Albano, 1980; Moschovakis et al., 1988a,b; Sparks, 2002a), have diverse subtypes (Sparks, 1978; Wurtz and Albano, 1980; Sparks and Hartwich-Young, 1989; Munoz and Wurtz, 1993a,b, 1995a,b), and are suggested to be more involved in cognitive and higher order functions (Everling et al., 1999; Horwitz and Newsome, 1999; Krauzlis et al., 2004, 2013; Sommer and Wurtz, 2004a,b; Dash et al., 2015).

It has been suggested that separation of sensory and motor events produces a transformation by activating separate circuits of cells to code different spatial variables (Pierrot-Deseilligny et al., 1991a,b; Gaymard and Pierrot-Deseilligny, 1999; Ohbayashi et al., 2003; Bays et al., 2010; Barber et al., 2013). Therefore, we hypothesized that the T-G transformation might occur as a transition of information through different cell types (V, VM, M) with fixed spatial codes. However, when the spatial tuning of our cells was examined at a more detailed time scale (Figs. 6, 7), this notion did not hold up. Instead, all three cell populations (V, VM, M) showed similar T-G transitions, consistent with extensive sharing of information along the dorsal-ventral layers of the SC (Massot et al., 2019). It therefore appears that the reason V cells mainly coded Te was simply because (by definition) they were more active early in the transformation, whereas M cells coded G because they were mainly active later in the response. Such dynamic codes have been demonstrated previously in VM cells in SC (Sadeh et al., 2015) and various brain areas during anti-saccades and double-step paradigms (Munoz and Everling, 2004; Zhang and Barash, 2004), but not in reactive saccades, and to our knowledge, not within cells that are primarily visual or motor.

However, different cell types clearly do not always share the same spatial code through time. For example, in the FEF we found that VM and motor cells coded different spatial attributes at the same time, i.e., at end of a memory delay (Sajad et al., 2016). At this time, it cannot be said whether this difference is due to the difference in brain structures, or different tasks. Further, based on our data we cannot exclude the possibility that some other cell classification scheme might better explain spatial coding in the SC, or that visual, VM, and motor cells might make different contributions to some other gaze task. However, it does appear that SC visual, VM, and motor cells share a time-dependent spatial transformation during reactive saccades.

### What produces the T-G transformation in reactive saccades?

When viewed as a spatiotemporal transformation (Figs. 6, 7), it became clear that the main determining factor for the SC spatial code during the reactive task was timing, and that this influence was distributed both within and across different cell types. The most likely explanation for this is that the SC is involved in a noisy, distributed sensorimotor transformation (Burns and Blohm, 2010; Franklin and Wolpert, 2011) that includes lateral and recurrent connections (Harting, 1977; Harting et al., 1980; Meredith and Stein, 1983; Fries, 1984; May, 2006). Given our current results, where might such noise arise during reactive saccades?

To consider this, it is important to note that the SC does not function in isolation, but instead has reciprocal connections to the FEFs, cerebellum, and thalamus, as well as feedback from the brainstem (Munoz and Guitton, 1985; Schall and Thompson, 1999; Optican and Quaia, 2002; Schall, 2002; Sommer and Wurtz, 2002). Thus, although we saw behavioral noise (i.e., in G) reflect in SC motor responses, we cannot assume that this is where they originated. Based on our results, we can reject the notion that noise only arose from a series of sequential transformations, because it was shared across different cell types. Nor could it arise from downstream noise that is not fed back to the SC, because this would equally degrade fits to both T and G rather than shifting T toward G (i.e., because uncorrelated downstream noise would dissociate SC activity from the final gaze behavior). However, it could result from (1) recurrent intrinsic connections within the SC (Gandhi and Katnani, 2011), (2) input from upstream sites like the FEF that influence all cell types (Sommer and Wurtz, 2002, 2004a; Marino et al., 2012), (3) feedback from downstream centers involved in saccade guidance (Sparks and Porter, 1983; Quaia et al., 1999), or (4) some combination of these. Each of these theoretical possibilities could be explored further by injecting noise at various points in a recurrent neural network model of the saccade generator. Finally, even our best spatial fits did not eliminate all residuals. This additional “noise” might arise from other unaccounted variables such as parallel sensorimotor pathways, attention, motivation, saccade latency, noise at the cell membrane level, or position dependences that did not meet our statistical detection thresholds (Sadeh et al., 2015).

### Clinical implications

Transforming sensory information to a movement command, even in the reactive saccade task, involves many different scenarios and task demands. It involves the integration of information and signals from various brain areas and for successful completion of a such seemingly simple task all of these components must be intact. Thus, the saccadic system can be viewed as a gateway for detecting abnormalities and a diagnostic tool in many neurologic and psychiatric disorders (Ketcham et al., 2003; Golla et al., 2008; Gooding and Basso, 2008; Redgrave et al., 2010; Terao et al., 2011). In this scenario, a major component of variable gaze errors results from the rapid accumulation and general spread of noise during the transformation from visual inputs to motor outputs. It is noteworthy, that we see this reflected in all of our SC cells. This noise is relative small during normal gaze shifts like those quantified here, but could become quite large during certain clinical conditions (Ketcham et al., 2003; Rottschy et al., 2013; Avery and Krichmar, 2015). For this reason, the analysis tools used here could be useful for providing clues about the source of sensorimotor function in the affected circuits. In the absence of technical limitations, it would be ideal to directly trace such noise to its source using electrophysiological recordings like those used here. But given the limitations of this technique in humans, it is likely more practical to record behavioral noise and correlate this to known brain disorders and/or damage that can be detected through neuroimaging techniques.

## Conclusions

To our knowledge, this is the first study to track the spatiotemporal code in SC cells during simple reactive saccades toward a briefly flashed target. Our results demonstrate a rapid VM transformation across all visual, VM, and motor cells, rather than a sequential relay of information between cells with fixed spatial codes. Since our method was based on the difference between target position and variable gaze-point errors, this in turn suggests that these errors accumulated across a distributed gaze circuit. We cannot say if these results generalize to other brain areas, tasks, and motor behaviors, but given the simplicity of our task, and the evolutionary conservation of SC function, and its connections, it seems likely that similar processes occur alone or in conjunction with other transformations in many other areas and behaviors. If so, it may be clinically useful to correlate behavioral variability to brain damage when sensorimotor transformations become noisy to a degree that is pathologic.

## Synthesis

Reviewing Editor: Christian Hansel, University of Chicago Decisions are customarily a result of the Reviewing Editor and the peer reviewers coming together and discussing their recommendations until a consensus is reached. When revisions are invited, a fact-based synthesis statement explaining their decision and outlining what is needed to prepare a revision will be listed below. The following reviewer(s) agreed to reveal their identity: NONE.

The remaining issues have been adequately addressed.
